# Reversal of Dabigatran with Idarucizumab in Acute Subarachnoid Hemorrhage

**DOI:** 10.5811/cpcem.2017.6.34356

**Published:** 2017-10-06

**Authors:** Jonathan Balakumar, Ruben Santiago, Mark Supino

**Affiliations:** Jackson Memorial Hospital, Department of Emergency Medicine, Miami, Florida

## Abstract

Dabigatran etexilate mesylate is a direct thrombin inhibitor used for reducing the risk of stroke and systemic embolism in patients with non-valvular atrial fibrillation. Dabigatran belongs to a new generation of oral agents for anticoagulation – the direct oral anticoagulants (DOACs). The DOACs also include the factor Xa inhibitors rivaroxaban, apixaban, and edoxaban. In the case of major or life-threatening bleeding and/or the need for emergent invasive procedures, a reversal agent is needed if a patient is taking one of these medications. Research has shown the efficacy of idarucizumab as an antidote in healthy volunteers, but data in the case of life-threatening bleeds remains limited. We report a case of a patient who suffered a traumatic subarachnoid hemorrhage and received effective treatment with idarucizumab. Along with other reports, our case demonstrates that dabigatran-related major and/or life-threatening bleeds may be effectively counteracted by idarucizumab. This provides an option to emergency department providers in managing clinically significant bleeds in patients taking dabigatran.

## INTRODUCTION

Dabigatran etexilate mesylate is a direct thrombin inhibitor used for reducing the risk of stroke and systemic embolism in patients with non-valvular atrial fibrillation. It is also indicated for the treatment or secondary prevention of venous thromboembolism.^1^ Dabigatran belongs to a new generation of oral agents for anticoagulation – the direct oral anticoagulants (DOACs). The DOACs also include the factor Xa inhibitors rivaroxaban, apixaban, and edoxaban. Compared to warfarin, observations from clinical trials and meta-analyses have suggested similar or lower major bleeding rates with the DOACs; however, in the case of major or life-threatening bleeding, and/or the need for emergent invasive procedures, a reversal agent is needed if a patient is taking one of these medications. Idarucizumab is the first antidote approved by the U.S. Food and Drug Administration (FDA) for the reversal of dabigatran.^2^ Research has shown the efficacy of idarucizumab as an antidote in healthy volunteers, but data in the case of life-threatening bleeds remains limited.^3^ Case reports have shown idarucizumab to be effective in emergency lumbar puncture procedures, acute subarachnoid hemorrhage, and reversing anticoagulation before administration of recombinant tissue plasminogen activator for ischemic stroke.^4^,^5^,^6^ We report a case of a patient who suffered a traumatic subarachnoid hemorrhage and received effective treatment with idarucizumab.

## CASE REPORT

An 86-year-old female presented to the emergency department (ED) after sustaining a head injury following a mechanical ground-level fall. Upon interviewing the family, it was determined that the patient was taking dabigatran. She had a left-sided blepharohematoma, left sided facial edema, blood on her lips, and a non-displaced fracture of the right mandible. Her medical history was significant for atrial fibrillation for which she was taking dabigatran 75mg twice daily with unknown timing of her last dose. Further history included hypertension, coronary artery disease, and a previous cerebral vascular accident. Pulse and blood pressure on admission were 118 beats per minute and 178/105 mm Hg respectively. She was alert and oriented to person, place, time, and situation, without focal neurological deficits. Her National Institutes of Health Stroke Scale was zero and her Glasgow Coma Scale was 15.

**Images 1:**
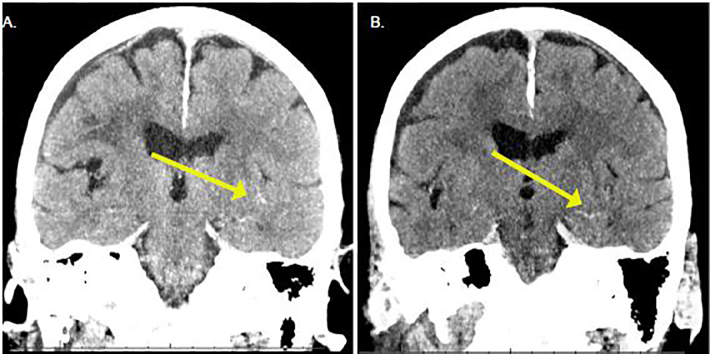
Coronal computed tomography demonstrating trace blood (arrows) at presentation **(A)** and four hours later **(B)** showing no progression of bleeding.

**Image 2:**
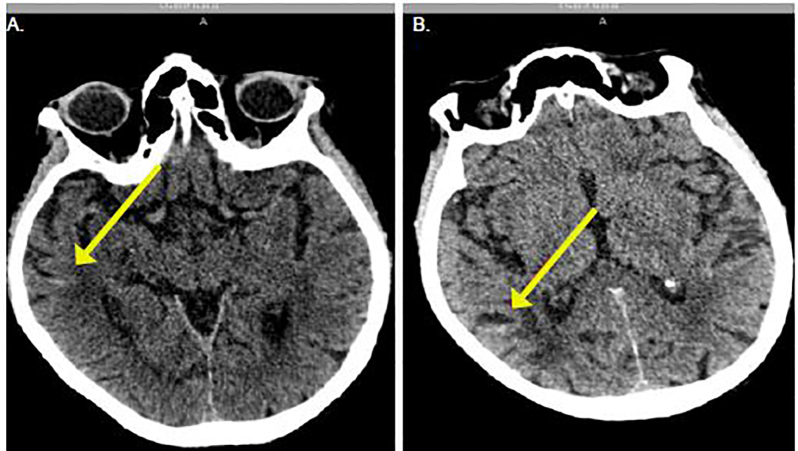
Axial computed tomography demonstrating trace blood (arrows) at presentation **(A)** and four hours later **(B)** showing no progression of bleeding.

The computed tomography (CT) showed signs of trace subarachnoid hemorrhage in the Sylvian fissures bilaterally and chronic bilateral subdural hygromas (Images 1 and 2). Laboratory findings showed an activated partial thromboplastin time (aPTT) of 30.8 seconds (s) (normal range 24.5–35.7 s), a mildly elevated prothrombin time (PT) of 12.7 s (normal 10.1–12.6 s) and a serum creatinine 1.17 mg/dL. The neurosurgery service, as well as the clinical pharmacy specialist for the ED, were consulted to initiate treatment with 5g of idarucizumab immediately to prevent further hemorrhage. Serial CT showed stabilization of the bleeding without further progression. Six days after admission the patient was transferred to a rehabilitation unit without incident.

## DISCUSSION

In October 2015, the FDA approved idarucizumab as the antidote for dabigatran when the reversal of the anticoagulant effect of dabigatran is necessary for situations such as emergency surgery, urgent procedure(s), or life-threatening or uncontrolled hemorrhage. A recombinant immunoglobulin G1 isotype molecule, idarucizumab is a fully humanized monoclonal antibody fragment that binds to the thrombin-binding site of dabigatran, resulting in the inability of dabigatran to bind to thrombin, ultimately neutralizing dabigatran’s anticoagulant effect. The affinity of idarucizumab for dabigatran is approximately 350-fold stronger than the affinity of dabigatran for thrombin.^7^,^8^ Idarucizumab is eliminated renally, but its reversal of the anticoagulant effects of dabigatran is not affected by renal function.^9^

**CPC-EM Capsule**

What do we already know about this clinical entity?

Subarachnoid hemorrhage is a bleeding between the arachnoid membrane and the pia mater that can be life- threatening and can result in poor neurological sequelae.

What makes this presentation of disease reportable?

This case demonstrates a traumatic subarachnoid hemorrhage in a patient taking dabigatran that was effectively treated with its antidote idarucizumab.

What is the major learning point?

Dabigatran can be treated with idarucizumab and effectively prevent further progression of intracranial bleeding in clinical practice.

How might this improve emergency medicine practice?

This provides an option to emergency department providers in managing clinically significant bleeding in patients taking dabigatran.

Although it has structural features similar to thrombin, idarucizumab does not bind to thrombin substrates, does not affect platelet aggregation, and does not possess thrombin-like enzymatic activity. The recommended dose of idarucizumab is 5 g, administered as two consecutive intravenous infusions of 2.5 g in 50 mL, no more than 15 minutes apart. Monitoring for dabigatran is not routine; however, it may increase coagulation tests such as thrombin time (TT), aPTT, international normalized ratio, ecarin clotting time (ECT), and dilute thrombin time (dTT). In the research setting, these assays may be used to evaluate bleeding while on dabigatran and monitor the use of idarucizumab; however, these assays may not be routinely available in the clinical setting.^7^,^8^ Idarucizumab has been shown to promptly reverse the effects of dabigatran in individuals with serious bleeding or those who require urgent invasive procedures.^10^

In an interim analysis of a phase III, ongoing, multicenter, prospective cohort study of the Reversal Effects of Idarucizumab on Active Dabigatran (RE-VERSE AD), results of the first 90 patients were reported. The study included two distinct groups: group A (51 patients) were patients with overt, uncontrollable, or life-threatening bleeding; and group B (39 patients) were those who required surgery or other invasive procedure(s) that could not be delayed for more than eight hours. The primary endpoint was the maximum percentage of reversal of the anticoagulant effect of dabigatran as assessed by dTT or ECT within four hours after the complete administration of 5 g of idarucizumab. Idarucizumab was administered as two 2.5 g 50 mL bolus infusions, no more than 15 minutes apart. A majority of the patients were taking dabigatran for atrial fibrillation at a dose of 110 mg twice daily. The median plasma levels of dabigatran at baseline were at therapeutic levels prior to the administration of idarucizumab. After the first vial of idarucizumab, the unbound dabigatran concentration was less than 20 mg/mL, a level that produced little or no anticoagulant effect in all but one patient and was maintained for 24 hours in 62 of 78 (79%) available blood samples. Normalization of dTT was achieved in 98% and 93% of patients that could be evaluated in group A and group B, respectively. ECT was normalized in 89% and 88% of patients that could be evaluated in group A and group B, respectively. Median time to cessation of bleeding in group A as determined by the investigator was 11.4 hours. Normal intraoperative homeostasis was reported in 92% of patients in group B. Overall there were 18 deaths (nine in each group) and five thrombotic events (three venous thromboembolisms, one non-ST segment myocardial infarction, and one ischemic stroke).^10^

Updated results to the RE-VERSE AD trial were presented in 2016. This contained data from123 patients (group A: 66, group B: 57). After 5 g of idarucizumab infusion, complete reversal of dabigatran was achieved in greater than 89% of patients. In 48 assessable patients in group A, median time to cessation of bleeding was 9.8 hours. In group B, mean time to surgery was 1.7 hours after infusion with a normal intraoperative homeostasis reported in 48/52 assessable patients. A total of five patients experienced a thrombotic event two to 24 days post infusion. Of the 123 patients, 26 died either due to comorbidities or worsening of their emergency condition.^11^

While the results of the RE-VERSE AD trial are promising, there have been case reports with various outcomes reported in the literature. In a report by Alhashem et al., a 65-year-old male, who was taking dabigatran for atrial fibrillation, presented with a chief complaint of generalized weakness and shortness of breath. On examination, the patient had a gastrointestinal hemorrhage and vital signs significant for an irregular heart rate at 122 beats per minute, and a blood pressure of 74/52 mm Hg. The patient received packed red blood cells (PRBCs) along with an infusion of 5 g of idarucizumab. After visualization of a hemorrhaging vessel through an esophagogastroduodenoscopy, numerous attempts to control the bleeding were unsuccessful. The patient remained critically ill resulting in the administration of clotting factor concentrate (factor eight inhibitor bypassing activity), additional PRBCs, and emergency angiography. He was discharged on hospital day 4.^12^

Marino et al. reported a case of a 58-year-old woman with a history of atrial fibrillation for which she was taking dabigatran 150 mg twice daily. This patient developed acute kidney injury that resulted in coagulopathy. The patient received an infusion of idarucizumab 5 g and hemodialysis. Despite these interventions, there were rebound increases in PT and aPTT values, prompting administration of another idarucizumab 5 g and continued dialysis. PT and aPTT values remained in their appropriate ranges after this intervention. The patient was formally diagnosed with end-stage renal disease and was placed on dialysis.^13^

In a case report by Peetermans et al., a 68-year-old female attempted suicide by ingesting 125 capsules of 150 mg of dabigatran. Despite gastric lavage and administration of activated charcoal, the aPTT and PT remained prolonged. After administration of idarucizumab 5 g, the patient’s aPTT decreased from 75 s to 26 s and PT decreased from 26 s to 11 s. The patient was discharged home with psychiatric follow-up.^14^

A report by Thorborg and colleagues describes a 79-year-old female who presented to a community hospital with abdominal discomfort. She was taking dabigatran 110 mg twice daily for atrial fibrillation along with clopidogrel 75 mg once daily. Upon imaging, the patient showed rectal perforation and peritonitis and was taken to surgery. There was severe derangement of coagulation as evident through whole blood rotational thromboelasometry (ROTEM). She received antifibrinolytics and aggressive blood product support. Idarucizumab 5 g was infused resulting in rapid cessation of hemorrhage and complete normalization of ROTEM. Significant anticoagulant activity and bleeding reoccurred with elevated dabigatran concentrations. Due to the patient’s declining status, a second dose of idarucizumab was not administered.^15^

In a report by Henderson et al., a 79-year-old male with a past medical history of atrial fibrillation for which he was taking dabigatran 150 mg twice daily, required emergency repair of an acute type-A aortic dissection from the aortic root to femoral arteries. The patient was given an infusion of idarucizumab 5 g prior to surgery to reverse the anticoagulant effects of dabigatran. Reversal of anticoagulant effects was evident on thromboelastography (TEG). A challenging surgical course ensued, with a total cardiopulmonary bypass time of 345 minutes, including 23 minutes of hypothermic circulatory arrest. A repeat TEG during rewarming indicated severe coagulopathy. The weaning of cardiopulmonary bypass was unsuccessful despite multiple inotropic drugs, and the decision was made to withdraw care.^16^

We report a case of a traumatic subarachnoid hemorrhage in a patient receiving dabigatran 75 mg twice daily in which idarucizumab successfully antagonized the effects of dabigatran, preventing further progression of the subarachnoid hemorrhage. While laboratory parameters such as the aPTT were not appreciably prolonged, therapy was guided by the area of the bleed and was monitored with subsequent CT imaging. Discontinuing dabigatran, monitoring bleeding, and administering idarucizumab based primarily on bleeding rather than laboratory testing is suggested by the current Neurocritical Care Society and Society of Critical Care Medicine Guideline for Reversal of Antithrombotics in Intracranial Hemorrhage. If idarucizumab is not available, administration of clotting factor concentrates is recommended. Idarucizumab can also be re-dosed if intracranial hemorrhage persists in patients who have already received a dose of idarucizumab and clotting factor concentrates. Due to the limited availability of dTT and ECT, the manufacturer makes no recommendations currently regarding laboratory monitoring. As a result, there is no recommendation on monitoring or re-dosing reversal agents based on laboratory parameters. Re-dosing of idarucizumab should be based on evidence of clinically significant ongoing bleeding.^17^ When excessively high concentrations of dabigatran are present, such as that in overdose situations, repeat dosing may also be necessary. However, the safety and effectiveness of repeat treatment have not yet been established.^9^

## CONCLUSION

Our case report demonstrates that in the presence of subarachnoid hemorrhage, with the patient neurologically intact, early and aggressive management with idarucizumab prevented further progression of the bleed. This provides an option to ED providers in managing clinically significant bleeds in patients taking dabigatran. While our patient did not experience any adverse effects with administration of idarucizumab, further research should continue to explore the safety and efficacy of this agent.
